# Ultrasound-Guided Greater Occipital Nerve Blocks and Pulsed Radiofrequency Ablation for Diagnosis and Treatment of Occipital Neuralgia

**DOI:** 10.5812/aapm.10985

**Published:** 2013-09-01

**Authors:** Matthew David VanderHoek, Hieu T Hoang, Brandon Goff

**Affiliations:** 1 Department of Anesthesia and Operative Services, San Antonio Military Health System, San Antonio Military Medical Center, San Antonio, USA; 2 Department of Orthopaedics and Rehabilitation, San Antonio Military Health System, San Antonio Military Medical Center, San Antonio, USA

**Keywords:** Pulsed Radiofrequency Treatment, Headache Disorders, Pain, Ultrasonography, Nerve Block

## Abstract

Occipital neuralgia is a condition manifested by chronic occipital headaches and is thought to be caused by irritation or trauma to the greater occipital nerve (GON). Treatment for occipital neuralgia includes medications, nerve blocks, and pulsed radiofrequency ablation (PRFA). Landmark-guided GON blocks are the mainstay in both the diagnosis and treatment of occipital neuralgia. Ultrasound is being utilized more and more in the chronic pain clinic to guide needle advancement when performing procedures; however, there are no reports of ultrasound used to guide a diagnostic block or PRFA of the GON. We report two cases in which ultrasound was used to guide diagnostic greater occipital nerve blocks and greater occipital nerve pulsed radiofrequency ablation for treatment of occipital neuralgia. Two patients with occipital headaches are presented. In Case 1, ultrasound was used to guide diagnostic blocks of the greater occipital nerves. In Case 2, ultrasound was utilized to guide placement of radiofrequency probes for pulsed radiofrequency ablation of the greater occipital nerves. Both patients reported immediate, significant pain relief, with continued pain relief for several months. Further study is needed to examine any difference in outcomes or morbidity between the traditional landmark method versus ultrasound-guided blocks and pulsed radiofrequency ablation of the greater occipital nerves.

## 1. Introduction

Occipital neuralgia is a condition manifested by chronic occipital headaches and is thought to be caused by irritation or trauma to the greater occipital nerve (GON). Characteristic headaches involve paroxysmal shooting or stabbing pain originating in the suboccipital region and radiating over the cranial vertex (1). Traditionally, the syndrome has been conservatively treated by medications, such as nonsteroidal anti-inflammatory drugs, tricyclic antidepressants, anticonvulsants and opioids. More invasive treatment methods, including Botox injections, acupuncture, implanted nerve stimulators, and pulsed radiofrequency ablation (PRFA) have emerged as treatment options for occipital neuralgia resistant to conservative treatment (2-4). GON blocks are one such method and have been used as the standard diagnostic and therapeutic tool for treating headaches thought to be caused by occipital neuralgia. GON blocks usually consist of injecting a mixture small amount of local anesthetic and steroid in proximity to the GON nerve. The greater occipital nerve is a branch of the second cervical nerve that runs along the posterior neck and scalp. It courses superiorlaterally where, at the level of the inion (occipital protuberance), it pieces the trapezius muscle and joins the occipital artery, which lies lateral to the nerve. There are, however, anatomic variations in its course [5]. Traditionally, GON blocks and PRFA have been performed using landmarks to locate the nerve and avoid injection into the nearby occipital artery (4-6). To utilize landmarks to guide the block, the occipital artery is palpated on the posterior aspect of the head at or slightly caudal to the level of the inion. Following a skin wheal with local anesthetic, a combination of long-acting local anesthetic and steroid (if desired) is injected in a field block manner just medial to the occipital artery pulse. An alternative landmark-guided method involves palpation of the inion and injection several centimeters lateral and inferior to the inion. Effort is made to avoid the occipital artery, which lies lateral to the nerve.

Ultrasonography offers an attractive alternative to using landmarks to guide injections and blocks of various different nerves and has been increasingly used both in the operating theater and pain medicine clinics to locate various soft tissues and track needle advancement under real-time visualization (7). There have been several studies examining the utility of ultrasound in occipital nerve stimulator implantation and in cadaveric GON blocks (8, 9). One study displayed that ultrasound-guided GON blocks may have better short-term efficacy than the landmark-guided method (10). These studies show ultrasound as a promising method to perform diagnostic blocks or PRFA of the GON in live patients. In this report, we describe two examples in which ultrasonography was used to guide diagnostic GON blocks and to direct PRFA of the GON for treatment of chronic occipital neuralgia.

## 2. Case Presentation

### 2.1. Case 1

**Figure 1.**
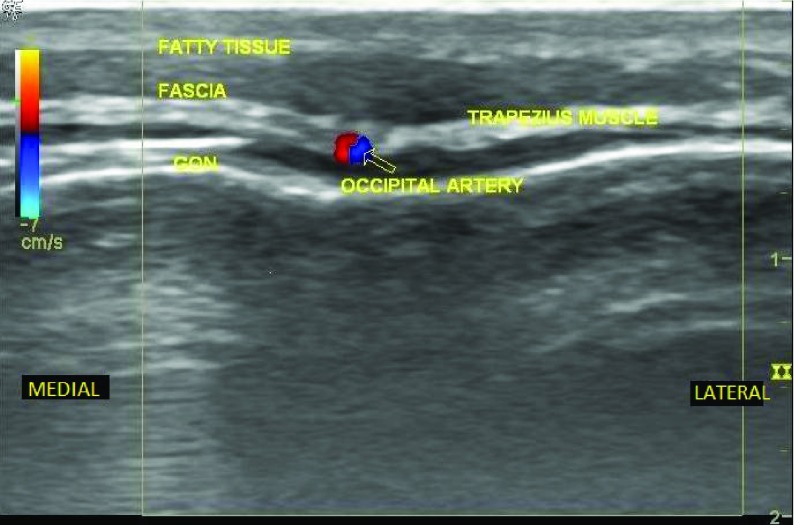
Patient 1; Ultrasound Image of the Greater Occipital Nerve in Relation to the Occipital Artery, Fatty Tissue, Fascia, and the Trapezius Muscle. GON = Greater occipital nerve

**Figure 2.**
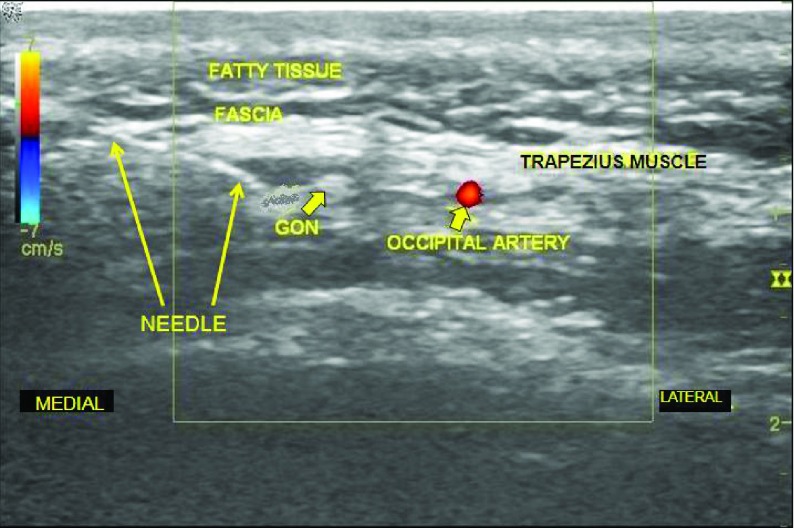
Ultrasound Image Showing the Needle Adjacent to the Greater Occipital Nerve Immediately Before Injection of Local Anesthetic/Steroid Mixture During Ultrasound-Guided Diagnostic Greater Occipital Nerve Block. GON = Greater Occipital Nerve

The first patient is a 35 year-old male active duty United States Army Soldier with chronic occipital headaches following several blast exposures between 2004 and 2006 from improvised explosive device (IED) explosions. His medical history includes postconcussive syndrome, essential hypertension, obstructive sleep apnea, post-traumatic stress disorder, and chronic low back pain. His pain from headaches ranged from 3-7 on a scale of 1-10 (1 being no pain and 10 being the worst pain he can imagine), with the headaches occurring three times per week. They were associated with photophobia, fatigue, and intermittent dizziness. Traditional pharmacotherapies were unsuccessful in improving the headaches. The patient was evaluated by behavioral health and his primary care physician and referred to our pain clinic for evaluation and treatment of his chronic headaches. To determine if occipital neuralgia was the cause of his headache, we proceeded with diagnostic bilateral greater occipital nerve blocks under ultrasound guidance. After informed consent, the patient was taken to the clinic procedure room, placed in the prone position, and the skin was then cleansed. Lidocaine 1% in a 27-gauge, 1 ½ inch needle was used to anesthetize the skin. Using a linear, high frequency ultrasound probe, the occipital artery and the greater occipital nerve were visualized (Figure 1). A 22-gauge, 1 ½ inch needle was advanced in the plane of the ultrasound beam until the tip was adjacent to the GON (Figure 2). Following aspiration that was negative for blood and cerebral spinal fluid, a mixture of 20 milligrams methylprednisolone and 2.5 milligrams bupivacaine 0.5% (1 milliliter total volume) was injected with visual confirmation of fluid spread around the target. The procedure was then repeated on the opposite side. The patient tolerated the procedure well, without bleeding, infection, or intraprocedural pain. Nearly all of the patient’s pain was relieved immediately post-block. The patient received therapeutic GON blocks every 4 – 6 weeks for treatment of occipital neuralgia that was diagnosed with the initial ultrasound-guided GON blocks before moving out of the area serviced by our pain management clinic. 

### 2.2. Case 2

The second patient is a 32-year-old male active duty United States Army Soldier with occipital neuralgia that had been previously successfully treated with several traditional GON blocks (landmark-guided) that provided pain relief for 2-3 months apiece. He also underwent a traditional bilateral GON pulsed radiofrequency ablation (also landmark-guided) with pain relief for 4-6 months. Other pertinent history includes chronic neck pain without radiculopathy and post-traumatic stress disorder. Due to the success of the prior pulsed radiofrequency ablation, a PRFA of the bilateral greater occipital nerves was planned, this time under ultrasound guidance.

Using a linear, high frequency ultrasound probe, the GON and occipital artery were visualized. First one and then a second standard radiofrequency probe needle with 10 millimeter active tips was advanced in-plane with the beam until adjacent to the GON on either side. PRFA was performed twice at each site with a rate of 2 Hertz and duration of 20 milliseconds for 120 seconds at a temperature between 38–42 degrees Centigrade. The procedure was tolerated well without complications and his occipital neuralgia was completely relieved for several months.

## 3. Discussion

To our knowledge, this is the first report of a diagnostic ultrasound-guided GON block or PRFA in a living subject. GON blocks and PRFA traditionally have been performed using palpation of anatomic landmarks such as the occipital protuberance and occipital artery pulse. With use of ultrasonography, it is possible to examine the precise location of both the GON and occipital artery as well as visualize, in real time, the advancement of needles used for GON blocks and PRFA. This can potentially avoid unnecessary trauma to surrounding tissue and structures, including implanted hardware such as a ventriculoperitoneal shunt. Also, local anesthetic infiltration around the GON during a block can be confirmed rather than using a blind technique. Jae et al. demonstrated that utilizing ultrasound to guide GON blocks for occipital headache treatment may yield improved pain scores in short term follow up. They theorize that this may be due to a more targeted approach to blocking the GON, rather than a field block, which is the technique used for the landmark-guided method (10). We believe ultrasonography to be extremely useful because it may increase the reliability of a diagnostic GON block in that visual confirmation of injectate around the target would further strengthen a diagnostic block that is ruled negative for occipital neuralgia. Perhaps, PFRA, where accuracy is greatly desired, may be an even more important use of ultrasound for treatment of occipital neuralgia. Despite the potential that ultrasound may improve the accuracy of an attempted GON block or PFRA of the GON and due to the low complication rate of landmark-guided GON blocks, a study showing superior patient safety with ultrasound use would require an extremely large number of participants and has not been conducted. Thus, ultrasound has not yet been shown to improve patient safety over that of the landmark-guided method. This being said, as with other ultrasound-guided peripheral nerve blocks, there is a level of comfort the practitioner may feel when seeing, in real time, that local anesthetic is infiltrating the targeted tissues and not being injected into a blood vessel or directly into the nerve. However, as a case report by Shankar and Simhan documenting inadvertent intravascular injection during an ultrasound-guided stellate ganglion block shows, ultrasound guidance does not ensure the avoidance of intravascular injection (11). Ultrasound is widely available and, we believe, makes the procedures easier for the practitioner once an initial learning curve is overcome. We also feel that ultrasound utilization will not add significantly to the time required to perform a GON block. This technique should be particularly helpful in patients with difficult to palpate occipital artery pulses, altered anatomy, or implanted hardware. Although ultrasound may not improve the safety of a GON block, the diagnostic utility and therapeutic benefit may be improved over the landmark guided method. 

Further study is needed to examine any short term as well as long term differences in outcomes and morbidity between the traditional landmark method versus ultrasound-guided blocks and PRRA of the occipital nerves. Study is also needed to determine if ultrasound utilization yields a lower false negative rate of diagnostic GON blocks.
